# P02.102. Evaluating the role of expectancy during stress-reducing aromatherapy in healthy older adults

**DOI:** 10.1186/1472-6882-12-S1-P158

**Published:** 2012-06-12

**Authors:** I Fonareva, M Demidenko, B Oken

**Affiliations:** 1 Oregon Health & Science University, Portland, USA; 2 Portland State University, Portland, USA

## Purpose

Aromatherapy is a common CAM approach for stress reduction, but its effectiveness and mechanisms remain unclear. It has been suggested that any stress-reducing benefits of aromatherapy are due to placebo (expectancy) effects. This study evaluates expectancy as a potential mechanism underlying stress-reducing aromatherapy actions.

## Methods

To date, 54 participants (mean age 58.7, 82% female) were randomized to either: 1) lavender (stress-reducing aroma), 2) coconut (detectable placebo), or 3) water (non-detectable placebo). The detectable placebo group was used to assess aroma-mediated expectancy. Further, half of the participants in each group, along with their aroma, received a prime suggesting they are inhaling a powerful stress-reducing aroma. The prime was used to evaluate verbally-mediated expectancy. Participants completed a visit during which they experienced the assigned aroma while undergoing a stress battery. Before and after the stress battery, we assessed participants’ subjective stress-related measures, cognitive function, salivary stress biomarkers, and physiologic measures including EEG and ECG. ANOVAs were used to detect group differences with all outcomes reflecting post-stress percent change from baseline.

## Results

Preliminary data show that, after the stress battery, there was no difference between the three aroma groups on subjective stress ratings and salivary cortisol and alpha amylase profiles (all p’s > 0.1). However, participants randomized to perceptible aromas (lavender and coconut), after stress battery, had decreased negative affect score on Positive and Negative Affect Scale compared to those randomized to water (p = .01). Further, though there was no effect of aroma on post-stress performance on cognitive tasks. Participants receiving a prime regardless of aroma group had smaller increases in median reaction time on Simple Reaction Time task , p = .01. Expectancy and aroma effects on EEG and physiologic responses will also be discussed.

## Conclusion

Preliminary data suggest that both aroma-mediated and verbally-mediated expectancy might be important in aromatherapy actions.

